# Respiratory Health Effects of Exposure to Low-NO_x_ Unflued Gas Heaters in the Classroom: A Double-Blind, Cluster-Randomized, Crossover Study

**DOI:** 10.1289/ehp.1002186

**Published:** 2010-07-27

**Authors:** Guy B. Marks, Wafaa Ezz, Nathan Aust, Brett G Toelle, Wei Xuan, Elena Belousova, Carmen Cosgrove, Bin Jalaludin, Wayne T. Smith

**Affiliations:** 1 Woolcock Institute of Medical Research, Sydney, New South Wales, Australia; 2 University of Sydney, New South Wales, Australia; 3 University of New South Wales, Sydney, New South Wales, Australia; 4 Environmental Health Branch, New South Wales Health Department, New South Wales, Australia; 5 University of Newcastle, Newcastle, New South Wales, Australia

**Keywords:** children, nitrogen dioxide, randomized controlled trial, respiratory health effects, schools

## Abstract

**Background:**

There are long-standing concerns about adverse effects of gas appliances on respiratory health. However, the potential adverse effect of low-NO_x_ (nitrogen oxide) unflued gas heaters on children’s health has not been assessed.

**Objectives:**

Our goal was to compare the respiratory health effects and air quality consequences of exposure to low-NO_x_ unflued gas heaters with exposure to non–indoor-air-emitting flued gas heaters in school classrooms.

**Methods:**

We conducted a double-blind, cluster-randomized, crossover study in 400 primary school students attending 22 schools in New South Wales, Australia. Children measured their lung function and recorded symptoms and medication use twice daily. Nitrogen dioxide (NO_2_) and formaldehyde concentrations were measured in classrooms using passive diffusion badges.

**Results:**

NO_2_ concentrations were, on average, 1.8 times higher [95% confidence interval (CI), 1.6–2.1] and formaldehyde concentrations were, on average, 9.4 ppb higher (95% CI, 5.7–13.1) during exposure to unflued gas versus flued gas heaters. Exposure to the unflued gas heaters was associated with increased cough reported in the evening [odds ratio (OR) = 1.16; 95% CI, 1.01–1.34] and wheeze reported in the morning (OR = 1.38; 95% CI, 1.04–1.83). The association with wheeze was greater in atopic subjects. There was no evidence of an adverse effect on lung function.

**Conclusions:**

We conclude that classroom exposure to low-NO_x_ unflued gas heaters causes increased respiratory symptoms, particularly in atopic children, but is not associated with significant decrements in lung function. It is important to seek alternative sources of heating that do not have adverse effects on health.

There has been long-standing concern about the health consequences of indoor exposure to gas combustion products (e.g., Garrett et al. 1998; Jarvis et al. 1996, 1998; Kerkhof et al. 1999; Melia et al. 1979; Ng et al. 2001; Phoa et al. 2004; Pilotto et al. 1997; Ponsonby et al. 2001a; Wong et al. 2004). Most concern has focused on the respiratory effects of emissions of oxides of nitrogen, particularly nitrogen dioxide (NO_2_) (Bauer et al. 1986; Chauhan et al. 2003; Tunnicliffe et al. 1994), although not all studies have demonstrated adverse effects at the observed concentrations (Salome et al. 1996; Samet et al. 1993).

There have been two randomized controlled trials that contribute a higher level of evidence. One of these is a parallel-group, randomized, controlled trial of a home-heating intervention conducted in a cohort of New Zealand houses that were poorly heated, including 55% with unflued gas heaters and others with electric or other heating sources, and in which at least one child had asthma (Howden-Chapman et al. 2008). Children whose homes were insulated and fitted with an effective nonpolluting heater had less nocturnal cough and wheeze, fewer lower respiratory tract symptoms, and better self-assessed health status during winter than those in the control group who did not receive any intervention until the study was over. There was no difference in lung function. This study demonstrated an improvement in clinically relevant health outcomes attributable to this home modification intervention. However, it is not clear whether the change was due to the nature of the fuel source or the effectiveness of the heating. Although the active group had substantially lower NO_2_ concentrations in the living room (8.5 μg/m^3^ vs. 15.7 μg/m^3^, *p* < 0.001) and in the child’s bedroom (means 7.3 μg/m^3^ vs. 10.9 μg/m^3^, *p* < 0.001) than the control group, there was also a 0.57°C higher average temperature in the child’s bedroom and a 50% reduction in the proportion of nights in which the temperature fell below 10°C in the active intervention houses. One limitation of the study was the absence of any blinding, which would not have been feasible in a parallel-group study. Because the outcomes that changed were all subjective, this possible source of reporting bias needs to be considered. However, despite these limitations, this study does strongly support the conclusion that there will be health benefits for children with asthma from the use of nonpolluting, effective domestic heating sources in cold climates.

The other randomized controlled trial was conducted in Australian schools (Pilotto et al. 2004). It was also a parallel-group, randomized, controlled trial in which there was no attempt to blind participants in the active intervention group that they were receiving new heaters. The control classrooms were heated with unflued heaters. Among children with asthma in the randomized classrooms, those in the active intervention classrooms were less likely to report difficulty in breathing (during the day or night), chest tightness (during the day), and asthma attacks during the day. There was no difference in lung function or airway hyperresponsiveness. As with the New Zealand study, the parallel-group design, which did not allow blinding, leaves some concern about possible reporting bias for subjective outcomes.

In New South Wales (NSW), unflued gas heaters have been the principal form of heating used in schools for many years. In response to emerging concerns in the late 1980s, the NSW Department of Education embarked on program of replacing its existing older-style unflued gas heaters with a newly designed low-NO_x_ (nitrogen oxides) unflued gas heater (Bowin Lo-NO_x_; Bowin mfg Pty Ltd, Sydney, Australia) (McPhail et al. 1989). These are designed to conform to a 2-ng NO_2_ per J standard (Bowin mfg Pty Ltd). However, there are continuing concerns about potential hazards arising from these heaters.

There has been one published report of a laboratory-based assessment of emissions from low-NO_x_ unflued gas heaters (Brown et al. 2004). This study showed that the five tested heaters did emit NO_2_ and, in some cases, formaldehyde and carbon monoxide. However, because no old-style heaters were tested using the same protocol, this study did not allow assessment of the extent to which these low-NO_x_ heaters were qualitatively or quantitatively different, as claimed, from the older-style heaters. Hence, it is difficult to know whether this study validates the application of health data from the clinical studies conducted with the older-style heaters to the low-NO_x_ heaters.

In summary, there are real health concerns about indoor exposure to combustion products from unflued gas heaters. Two randomized control trials suggest that benefits may follow from replacement of these unflued gas heaters, but the design of these trials imposes some limitations on interpretation. Furthermore, the previous Australian trial included both older-style and low-NO_x_ gas heaters in the control group (Pilotto L, personal communication), and only very limited data are available about potential adverse effects of these newer type of gas heaters. Hence, we conducted a randomized, double-blind, crossover study comparing the respiratory health effects and air quality consequences of exposure to newer-style low-NO_x_ gas heaters with exposure to non–indoor-air-emitting flued gas heaters.

## Methods

The study was a three-period, cluster-randomized, controlled, crossover study conducted in primary school classes. Two conditions were compared: heating with a low-NO_x_ unflued gas heater and heating with a flued gas heater. Classrooms were fitted with both types of heaters. In each classroom the existing low-NO_x_ unflued gas heater was left *in situ*, and the flued gas heater was installed co-located with it in the classroom. Both heaters were surrounded with a screen so that those in the room would not be able to see which one was operating. During the study period either the low-NO_x_ unflued gas heater or the flued gas heater was in active mode for 1-week periods. The 6-week study period was arranged in three consecutive pairs of 2 weeks. For each classroom, the order of operation of the unflued and flued gas heaters was randomized separately for each pair of weeks. Hence, each classroom used a low-NO_x_ unflued gas heater for three 1-week periods and a flued gas heater for three 1-week periods. During this time the heaters were operated at the discretion of the class teacher, in the usual way, according to heating need. Further details about the installation and control of heaters, blinding of participants, and the randomization procedure are provided in the Supplemental Material (doi:10.1289/ehp.1002186).

The study was conducted in schools in the Blue Mountains, Southern Highlands, and Goulburn regions of NSW. These are elevated areas outside the Sydney metropolitan area. They were chosen because of their relatively cold climate and proximity to Sydney. Baseline assessments were conducted in June 2009, a run-in and pilot-testing week was conducted beginning 3 August 2009, and the 6-week study period began on 10 August 2009. The pilot-testing week was 1 week after the 2-week mid-winter break. August is the last month of winter in the southern hemisphere.

The trial was approved by the Human Research Ethics Committee of the University of Sydney and the NSW Department of Education and Training State Education Research Approvals Process. Parents gave written informed consent prior to participation of their child in the research. The trial was registered with the Australian and New Zealand Clinical Trials registry (ACTRN12609000610235; 22 July 2009).

### Study population

There was a two-stage selection process in which schools and classrooms were selected and then participants within the selected classrooms were recruited. Eligibility criteria for classrooms were that they were fitted with existing unflued gas heaters, that it was feasible to install a flued gas heater, and that the class using the selected room included 20 students in grades 4, 5, or 6. Principals of 38 schools in the Blue Mountains, Southern Highlands, and Goulburn regions were invited to nominate classes to participate. All students in the selected classrooms were eligible for inclusion in the study. Those whose parents gave written informed consent were recruited.

### Monitoring heater use

Temperature monitors (HOBO H8 series data loggers; Onset, Pocasset, MA, USA) were installed in the enclosures to estimate the running times for each heater. Abrupt changes in temperature were used to identify the times when heaters were turned on or off. These temperature loggers began operating only from the third study week (24 August 2009).

In-class temperature was also recorded at 2-min intervals between 0900 and 1500 hours on each study day.

### Respiratory health assessments

We collected baseline information on respiratory symptoms and illnesses and use of gas and other heaters at home using a questionnaire completed by the parents. We also measured spirometric lung function before and after administration of 200 μg albuterol via spacer. Current asthma was defined as reported wheeze in the preceding 12 months and either a reported doctor’s diagnosis of asthma or an increase in forced expiratory volume in 1 sec (FEV_1_) after albuterol that was ≥ 12% of baseline. Height and weight were measured using a stadiometer and portable scales, respectively.

We measured atopy by using skin prick tests to inhalant allergens *Dermatophagoides pteronyssinus* (HDM), *Dermatophagoides farina* (also HDM), cockroach, cat, *Alternaria*, *Aspergillus*, rye grass, and a grass mix (Hollister-Stier, Spokane, WA, USA), as previously described (Peat et al. 2004). Glycerol and histamine phosphate 10 mg/mL were used as negative and positive controls, respectively. Wheal sizes were measured 15 min later and those that were ≥ 3 mm and were also greater than negative control were classified as positive. Subjects with positive reactions to any allergen were classified as atopic.

The main study outcomes were symptoms and lung function measurements recorded twice daily by participants. Children kept a daily symptom and medication diary for the 6-week study period. Subjects were asked to record each morning the presence of cough and wheeze or chest tightness during sleep. They were asked to record each evening the presence of cough, wheeze or chest tightness, fever, sore throat, runny or blocked nose, eye irritation, sore teeth or gums, and stomachache during the day. The last two questions were included to detect possible information bias. For each symptom subjects were asked to indicate whether they had “no symptoms during the day” (scored 0), “symptoms, but did not disturb your daily activities” (score 1), “symptoms that disturbed part of your daily activities” (score 2), or “symptoms that disturbed the whole day or most of your daily activities” (scored 3). Subjects also recorded whether they used bronchodilator (reliever) medication twice a day and, if so, whether it was for relief of symptoms or as part of their regular medication regimen. In the evening subjects also recorded if they had taken preventer medications for asthma, been exposed to an unflued gas heater or other type of heater at home, and/or been exposed to environmental tobacco smoke (ETS) during that day.

We used Mini-Wright Digital (MWD) portable electronic spirometers (Clement Clarke, Harlow, Essex, UK) to record lung function twice daily. Children were instructed to perform three forced expiratory maneuvers into their MWD devices in the morning and in the evening. The device records only the highest FEV_1_ and peak expiratory flow rate (PEF) from the attempts made during any session. Each recording is made with an associated date and time stamp. Detailed instructions on how to do this were included on the reverse side of the diary card.

Each week the diaries were collected, and MWD data were downloaded during the school visits. The parents were sent a text message (SMS) the day before the teams were in the schools to remind the children to bring the diaries to the schools the next day. After the diaries were collected they were checked for errors and missing information. Families were then telephoned to attempt to correct the mistakes or to fill in the missing information.

Once each week, on Thursday or Friday, we recorded PEF and FEV_1_ using the child’s own MWD device. This served as an in-school measurement of lung function and also provided an opportunity to check their technique and correct it if needed. In addition at these school visits, we also recorded exhaled nitric oxide (eNO), using the offline method (Salome et al. 1999).

### Measurement of indoor NO_2_ and formaldehyde concentrations

Airborne NO_2_ concentrations in school classrooms were measured using passive diffusion badge monitors supplied by the CSIRO Division of Atmospheric Research; Clayton South, Victoria, Australia). Formaldehyde samples were collected using UMEx 100 (SKC Inc., Eighty Four, PA, USA) passive sampling badges.

On Thursday and Friday of every week during the study period, two NO_2_ samplers were exposed at two different locations in each classroom. One formaldehyde sampler was co-located at one of these sites on both occasions. Further details about the placement of the samplers and their chemical analysis are provided in the Supplemental Material (doi:10.1289/ehp.1002186).

### Study power and sample size estimates

On the basis of previous data from our research group, we estimated that the within-subject SD in FEV_1_ was 200 mL. On this basis, using a simple crossover paired *t-*test analysis, 43 subjects would be sufficient to give 90% power to detect difference in FEV_1_ between the two exposures of ≥ 100 mL. We expected to have an average of 15 student participants in each class. If the intraclass correlation coefficient for classrooms was as high as 0.2, then the design effect would be 3.8 and the required sample size increased to 164 subjects in 11 classrooms. To contend with potential nonparticipation and dropout among subjects and to facilitate the subgroup analyses proposed in the second hypothesis, we intended to recruit an average of 20 students from each of 20 classrooms, for a total of 400 children.

### Statistical analysis

Analysis was conducted in SAS 9.2 (SAS Institute Inc., Cary, NC, USA). A *p*-value (alpha) < 0.05 was regarded as statistically significant.

A mean morning symptom score and a mean evening symptom score (excluding “sore teeth or gums” and “stomachache”) was calculated for each subject for each day. PEF and FEV_1_ recordings made between midnight and 1200 hours were designated as morning values, and those made between 1200 hours and midnight were designated as evening values. We limited the data analyzed to those relevant to a period within 24 hr after the last exposure to heaters in schools. Hence, analyses of morning symptoms and lung function data excluded observations made on Sunday and Monday mornings, and analyses of evening reports of symptoms and records of lung function excluded observations made on Saturday and Sunday evenings.

The effect of randomized heater type allocation (that is, exposure to flued vs. unflued gas heaters) was estimated using hierarchical or mixed-model regression. Fixed effects were the heater type allocation, study period (first, second, or third pair of weeks), and day of the week. In a preliminary analysis we also included heater type randomization order (that is, flued heater in the first week of a pair vs. unflued heater in the first week of a pair) and the heater type by heater type order interaction to test for order effects. Subjects were included as random intercept assuming an unstructured (variance components) correlation matrix for the outcomes measured on individuals (diary card and lung function outcomes). Mixed models treat missing values as missing at random, which is a less-rigorous assumption than missing completely at random. School was included as either a random effect or a fixed effect, as described below.

In a planned subsidiary analysis, we tested for current asthma status by heater type and atopic status by heater type interactions; where these interactions were significant, we fitted separately the models described above for the subgroup with current asthma or the subgroup with atopy.

The primary analysis used all data in an intention-to-treat (ITT) analysis. However, we also fitted a per-protocol model in which the analysis was limited to schools and days for which heater use was estimated as ≥ 30%. We also tested the effect of three time-variable confounders—use of gas heaters at home, use of an open fire at home, and exposure to ETS at home—on the effect of the randomized heater–type exposure on symptoms. Each of these three confounding factors was measured daily using the diary.

For normally distributed continuous outcomes (FEV_1_, PEF, exhaled NO, and in-class formaldehyde), we used Proc Mixed (version 9.2; SAS Institute Inc., Cary, NC, USA) to fit the model assuming a normal error distribution. School was included as an additional random-intercept term in these models. We used a similar model for the log_10_-transformed values of in-class NO_2_ concentrations. The effect of heater type on the outcome was estimated as a difference with its 95% confidence interval (CI). The difference in log-transformed NO_2_ concentrations was raised to the power of 10 (anti-logged) to yield a ratio.

Symptoms were treated as both a binary variable (present or absent) and a continuous score variable (0–3) and analyzed using Proc GLIMMIX with the Laplace estimation routine (SAS Institute Inc.). For binary outcomes, we used a logistic link and binary error structure and estimated odds ratios (ORs). For score variables, we used a log link and Poisson error structure. In some cases it was not possible to fit the model when school was included as a second random intercept. Therefore, for all logistic and Poisson models, school was included as an additional fixed effect rather than a random effect.

## Results

### Study participants

Twenty-two schools participated in the project. Overall, 418 subjects participated in at least one part of the study, representing 77% of the 543 students in the selected classes. Three students did not submit a baseline questionnaire. At least one diary was received for 400 participants (74% of eligible students). These participants formed the study population. In any given week, average participation rates ranged from 64% to 73%.

Slightly over half the participants were girls, and their mean age was 10.6 years (Table 1). Nearly half were atopic, including 34% who were sensitized to house dust mite allergen. Approximately 15% had current asthma, but 21% were using some asthma-related medication. Unflued gas heaters were used in nearly one quarter of all participants’ homes and half used gas for cooking.

### Use of heaters in schools

Heater use was monitored during weeks 3–6 and was quantified as the proportion of time between 0900 and 1500 hours that the heaters were in operation. Across the 22 schools, median (interquartile range) daily heater use during weeks 3–6 was 25.7% (0–47.2%), 30.4% (6.9–47.8%), 16.7% (0–41.7%), and 0% (0–9.7%) of the school day, respectively. Heater use was recorded as ≥ 30% on 157 school days.

Mean daily in-class temperature between 0900 and 1500 hours was 20.9°C on days when the unflued gas heater was operating and 20.5°C on days when the flued gas heater was operating (difference adjusted for random effects 0.4°C; 95% CI, 0.0–0.8, *p =* 0.07). When the analysis was limited to days when the heaters were operating for at least 30% of the day, the mean temperatures were slightly higher during the period of operation of the unflued gas heater (19.6°C) than during the periods of operation of the flued gas heater (18.6°C). The mean difference was 1.0°C (95% CI, 0.5–1.5).

### Effect of heater type on NO_2_ and formaldehyde concentrations in the classroom

The overall geometric mean NO_2_ concentration in classrooms was 32 ppb (60.2 μg/m^3^), and the overall mean concentration of formaldehyde was 29 ppb (35.6 μg/m^3^) during school hours on the days of measurement.

There was a significant order by heater type interaction in the effect on NO_2_ concentrations (*p =* 0.01). However, as both order sequences showed an increase in NO_2_ levels when the unflued gas heater was operating, order effects were not considered further. The order by heater type interaction was not significant for formaldehyde.

There were highly significant effects of heater type on concentrations of NO_2_ and formaldehyde in the classrooms (Tables 2 and 3). The effect was larger in magnitude when the analysis was limited to weeks and classrooms in which heaters were used for ≥ 30% of the time.

### Estimated effects of heater type on lung function and airway inflammation (eNO)

The randomized order in which classrooms were exposed to the two types of heaters (unflued gas heaters and flued gas heaters) had no significant influence on the effects of the heater type on lung function or airway inflammation (*p* > 0.2 for all heater type by order interactions). However, the estimated effect of heater type on PEF measured in the evening differed between people with and without asthma (*p =* 0.01). Atopic status did not influence the effect of heater type on lung function or eNO concentrations (all *p* > 0.1).

In the ITT analysis for the whole study population, heater type did not have any significant effect on any of the lung function measures after adjustment for day of the week, study week, and clustering by subject and school (Table 4). However, when the analysis was limited to days and schools in which heater use was ≥ 30%, morning FEV_1_ was 0.03 L (95% CI, 0.003–0.057, *p =* 0.03) higher during the period of exposure to the unflued gas heater. Similarly, in this subgroup, evening PEF was 5.2 L/min (95% CI, 1.0–9.3, *p =* 0.01) higher during operation of the unflued gas heater. Evening PEF was 7.3 L/min (95% CI, 0.6–13.7) higher during the operation of the unflued gas heater in the ITT analysis in the asthma subgroup but not in the nonasthma subgroup (difference = −0.1 L/min; 95% CI, −2.2 to 1.9). In the per-protocol analysis (when heater use was ≥ 30%) for the asthma subgroup, the association of unflued gas heater exposure with higher evening PEF was similar but was not statistically significant.

The findings on lung function were not substantially different when data were excluded for subject days on which the subject used a bronchodilator in the morning or the evening [Supplemental Material, Table E1 (doi:10.1289/ehp.1002186)]. There was no evidence of any decrement in lung function attributable to unflued gas heater exposure when the analysis was restricted in this way.

Analysis of lung function measures recorded during in-school testing on Thursday or Friday of each study week revealed that the unflued gas heater was associated with a small increase in FEV_1_ [0.03 L (95% CI, 0.01–0.05), *p* = 0.01] compared with the flued gas heater. The magnitude and direction of the estimated effect on FEV_1_ was similar in the per-protocol analysis [0.03 L (95% CI, −0.01 to 0.08), *p =* 0.14]. There were no significant effects on PEF measured at the same time, although the trend was in the same direction (data not shown).

Overall, eNO levels, which were measured at the same time as the in-school lung function measurements, did not differ between periods of exposure to the two heater types in either the ITT or per-protocol analysis. However, there was a significant interaction with current asthma status in the per-protocol analysis (*p =* 0.002). In the subgroup with current asthma, eNO concentrations were 3.7 ppb higher during exposure to the unflued gas heater than during exposure to the flued gas heater (95% CI, −0.5 to 7.9, *p =* 0.08). However, in the subgroup without asthma, the effect of flued versus unflued gas heater exposure on eNO concentrations was close to zero [0.03 ppb (95% CI, −0.68 to 0.74), *p* = 0.93].

### Effect of heater type on respiratory symptoms

The overall daily prevalence of wheeze in the morning was 4.9% during periods of exposure to the low-NO_x_ unflued gas heater and 4.4% during exposure to flued gas heater (Table 5). For wheeze reported in the evening, the overall prevalence was 5.7% and 5.2% respectively. The prevalence of cough reported in the morning was 14.2% during both exposure periods, and the prevalence of cough reported in the evening was 21.6% and 20.6%, respectively.

Treatment order did not influence the effect of heater type on symptoms except in relation to symptoms recorded in the evening (*p =* 0.02). However, no separate analysis by treatment order was undertaken. The estimated effect of heater type on symptoms did not differ significantly by current asthma status, but did according to atopic status, particularly in relation to wheeze recorded in the morning (*p =* 0.0009) and in the evening (*p =* 0.01) and the use of bronchodilators to relieve symptoms (*p =* 0.002).

In the ITT analysis, exposure to the unflued gas heater was associated with an increase in cough reported in the evening and wheeze reported in the morning (Table 6). When the analysis was restricted to days when heater use was ≥ 30%, the findings were generally similar, although no significant differences were found. After adjustment for use of unflued gas heaters or open fires at home and exposure to ETS at home, the association of heater type with wheeze reported in morning was stronger (OR = 1.60; 95% CI, 1.17–2.19, *p =* 0.003) than without adjustment (OR = 1.38; 95% CI, 1.04–1.83, *p =* 0.03). However, the association with cough reported in the evening was weaker and no longer significant after adjusting for these confounders.

The association with morning wheeze was evident in atopic subjects in the ITT analysis (OR = 1.85; 95% CI, 1.26–2.72, *p =* 0.002) and was particularly strong in this subgroup when the analysis was limited to days when heater use was ≥ 30% (OR = 7.72; 95% CI, 1.55–38.43, *p =* 0.01). The evidence of an association with wheeze in atopic subjects was strengthened by the finding of an increased use of bronchodilators for relief of symptoms with exposure to the unflued gas heater in this subgroup (OR = 1.87; 95% CI, 1.08–3.25, *p =* 0.03). In the nonatopic subgroup, these adverse effects were not observed. In the ITT analysis in nonatopic subjects, there was no significant association with wheeze reported in the morning (OR = 0.63; 95% CI, 0.39–1.02, *p =* 0.06). There was a significant protective association with wheeze reported in the evening (OR = 0.50; 95% CI, 0.30–0.84, *p =* 0.009) and with the use of bronchodilator for relief of symptoms (OR = 0.45; 95% CI, 0.23–0.91, *p =* 0.03). The atopic subgroup was slightly less likely to report cough in the morning when exposed to the unflued gas heaters (OR = 0.77; 95% CI, 0.60–0.98, *p =* 0.03).

There was no consistent evidence of an association between heater type and reporting of sore teeth or stomachache, although the latter was associated with exposure to unflued gas heaters in the atopic subgroup in the ITT analysis (OR = 1.63; 95% CI, 1.06–2.49, *p =* 0.02).

The findings when symptoms were analyzed based on symptom scores (0–3) were broadly similar to those observed when symptoms were treated as simply present or absent (data not shown). The association between heater type and wheeze score differed significantly between atopic and nonatopic subjects, with more adverse associations in the atopic subgroup (*p =* 0.0008 for morning wheeze and *p =* 0.0001 for evening wheeze). No significant estimated effects of heater type on wheeze scores were observed in the overall ITT or per-protocol analyses. However, in the atopic subgroup, there was a significantly increased wheeze score in the evening during exposure to the unflued gas heater in the ITT analysis (*p =* 0.05), and this was more significant in the per-protocol analysis in this subgroup (*p =* 0.02).

### Number needed to treat

The absolute difference in daily prevalence of wheeze between when the low-NO_x_ unflued gas heater was scheduled to operate and days when the flued gas heater was scheduled to operate was 0.5% (4.9% vs. 4.4%). This is equivalent to a number needed to harm of 200 (1/0.005). Hence, 200 children need to be removed from exposure to low-NO_x_ unflued gas heaters during the 6-week period in which this study was conducted to prevent one child reporting wheeze in the morning on an average day.

## Discussion

We have shown that in-class exposure to unflued gas heaters is associated with *a*) increased reporting of wheeze in the morning and possibly with increased cough and wheeze during the day (reported in the evening) and *b*) in children with current asthma, an increase in airway inflammation, manifest as eNO. However, exposure to unflued gas heaters was not associated with a significant reduction in lung function, whether measured at home or at school. In fact, there was a paradoxical increase in evening PEF with exposure to unflued gas heaters when the analysis was restricted to days when heater use was ≥ 30% of school time and in subjects with asthma. The association of unflued gas heating with cough and wheeze was greatest in atopic (allergic) subjects, who represented over half the study population. Atopic subjects were also more likely to use bronchodilator (reliever) medications during exposure to unflued gas heaters. We have also shown that mean NO_2_ and formaldehyde levels were substantially increased in classrooms during the operation of unflued (compared with flued) gas heaters.

### Strengths and limitations

The major strengths of this study design are the randomized crossover design, the blinding of participants and researchers to exposure allocation, the inclusion of items designed to detect information bias, and the use of objective outcome measures. Taken together, these attributes give a high level of confidence that selection bias, confounding, and information bias have been avoided by design or would have been detected if present. The absence of consistent evidence of an adverse association with stomachache or sore teeth gives some confidence that the adverse effect on wheeze and cough was not attributable to information bias.

Another strength of the study is the high participation rate. This lends weight to the external validity of the study in relation to primary school children in NSW. The prevalence of asthma in this population (15.3%) is very close to the estimate for 9- to 15-year-olds in the recent NSW Health Survey (13.9%) (Centre for Epidemiology and Research 2010).

The main limitation of the study, as implemented, was the low usage of heaters during the study period, presumably because of the unseasonably warm weather in late August and early September. Temperature data for the three main towns in the study area are shown in Supplemental Material, Table E2 (doi:10.1289/ehp.1002186). This table shows that the temperatures increased toward the latter part of the study period. The lack of use of the heaters limited our capacity to assess their impact on children’s health.

Another limitation, which is inherent in this crossover study design, is inability to assess impacts on long-term health outcomes. Only short-term (acute) effects can be measured. However, studies designed to assess long-term effects are likely to have problems with selection bias and confounding, because it is not feasible to adopt a randomized design as we have in this study.

### Discordance between lung function and symptom findings

The discordance between the symptom and lung function data was unexpected, although it is consistent with the findings of the two previous randomized controlled trials in this field (Howden-Chapman et al. 2008; Pilotto et al. 2004). One possible explanation is that subjects with asthma used additional bronchodilators to relieve symptoms and that this resulted in an increase in their lung function at the time the measurements were made. The finding of increased reliever use in people with asthma tends to support this explanation.

An alternative explanation is that exposure to the unflued gas heater emissions results in sensory changes without effects on airway function. Indeed, there is evidence that NO_2_ does have effects on sensory neuropeptides in the respiratory tract in an animal model (Lucchini et al. 1996).

### Sensitive subgroups

There is evidence that NO_2_ exposure attributable to traffic is associated with an increase risk of wheezing illness in children (Pershagen et al. 1995; Peters et al. 1999) and an increased risk of hospitalizations for asthma (Simpson et al. 2005). Hence, we expected that children with asthma would be more susceptible to the adverse effects of exposure to unflued gas heaters. In fact, the findings do not generally support this conclusion.

We did find that atopic status defined a large subgroup of the population who were more likely to report symptoms with exposure to unflued gas heaters. This is consistent with the observation that pre-exposure NO_2_ was associated with increased early and late asthmatic response to house dust mite allergen challenge in sensitized subjects (Tunnicliffe et al. 1994). However, in that study, this effect was seen only at high levels of exposure (400 ppb) and not at 100 ppb. There is also evidence from some epidemiological studies that the effects of exposure to gas appliance were more marked in atopic subjects (Corbo et al. 2001; Garrett et al. 1998; Jarvis et al. 1996; Kerkhof et al. 1999), although others have not observed this result (Ponsonby et al. 2001b).

## Conclusion

We conclude that, when compared with exposure to flued gas heaters, classroom exposure to newer-style low-NO_x_ unflued gas heaters increased respiratory symptoms, particularly in atopic children, but was not associated with measurable adverse changes in lung function. Although this latter finding reassures us that severe adverse consequences are unlikely in the short term, the increased burden of symptoms and the observation of higher levels of eNO with exposure to the unflued gas heater in the subgroup with asthma suggest an adverse effect that is best avoided. It is important to seek alternative sources of domestic and public space heating that do not have adverse effects on health but are also effective and efficient for heating and have a favorable environmental profile.

## Figures and Tables

**Table 1:** Baseline characteristics of the study population that completed at least one diary card.

Characteristic	*n*^a^ (%) or mean ± SD
Total subjects with diary cards	400 (100)
Females	220 (55)
Atopic^b^	
Yes	152 (44)
No	192 (56)
Missing	56
Wheeze ever	168 (42)
Wheeze in the last 12 months	84 (21)
Doctor-diagnosed asthma	108 (27)
Positive bronchodilator test^c^	
Yes	39 (10)
No	337 (90)
Missing	24
Current asthma^d^	60 (15)
Use of any medicines for asthma	81 (21)
Smoking inside the house	28 (7)
Gas heating at home^e^	
Flued	66 (17) [9 missing]
Unflued	89 (23) [11 missing]
Gas for cooking at home	202 (51)
Age (years)	10.6 ± 0.9
Height (cm)	143.7 ± 8.8 [10 missing]
Weight (kg)	39.8 ± 10.4 [10 missing]
FEV_1_ (L)	2.02 ± 0.39 [12 missing]
FEV_1_ (% predicted)	90.8 ± 10.2 [16 missing]
Forced vital capacity (L)	2.39 ± 0.47 [12 missing]
Forced vital capacity (% predicted)	94.6 ± 10.5 [16 missing]
eNO (ppb)^f^	8.04 (2.3–27.7) [56 missing]

a Complete data or missing four or fewer subjects unless otherwise indicated.

b Sensitized to one or more of the test allergens.

c Increase in FEV_1_ after bronchodilator ≥ 12% of baseline value.

d Current asthma defined as wheeze in the last 12 months and either doctor-diagnosed asthma or a positive bronchodilator test.

e Subjects who indicated they had no heating or had another type of heating at home but who did not answer the question about gas heating were assumed not to have a gas heater. Subjects who had a flued gas heater were assumed not to have an unflued gas heater.

f Geometric mean and geometric 95% range are shown.

**Table 2:** Concentrations of NO_2_ and formaldehyde measured by badges, by treatment group.

	Overall		Flued		Unflued	
Gas (ppb)	Mean	95% Geometric range	Mean	95% Geometric range	Mean	95% Geometric range
NO_2_^a^	23.5	4.6–121.4	17.5	3.5–88.4	31.6	7.4–135.2
Formaldehyde	28.6	1.8–55.4	24.7	3.3–46.1	32.6	3.1–62.1

a Values for NO_2_ are geometric mean and geometric 95% range.

**Table 3:** Effect of heater type on NO_2_ and formaldehyde levels, measured by badges.

	NO_2_ (relative difference: unflued/flued)				Formaldehyde (absolute difference: unflued – flued ppb)			
Population	*n*	Mean ratio	95% CI	*p-*Value	*n*	Mean difference	95% CI	*p-*Value
ITT analysis^a^	248	1.80	1.55–2.10	< 0.0001	167	9.6	5.9–13.3	< 0.0001
Per protocol^b^ (weeks when heater use is ≥ 30%)	51	2.33	1.53–3.55	0.0002	44	20.1	10.6–29.6	0.0001

The ratios and differences and their 95% CIs are derived from a mixed-model regression. Fixed effects were the heater type allocation, study period (first, second, or third pair of weeks), and day of the week. Schools were treated as random intercept, and a variance components correlation structure was assumed.

a All observations are analyzed according to the heater type exposure to which they were assigned.

b The analysis was limited to schools and days for which heater use was estimated as ≥ 30%.

**Table 4:** Effect of heater type (unflued gas heater – flued gas heater) on lung function.^a^

	FEV_1_ morning (L)					FEV_1_ evening (L)					PEF morning (L/min)					PEF evening (L/min)				
Population	Unflued	Flued	Diff	95% CI	*p-*Value	Unflued	Flued	Diff	95% CI	*p-*Value	Unflued	Flued	Diff	95% CI	*p-*Value	Unflued	Flued	Diff	95% CI	*p-*Value
ITT analysis^b^	1.95	1.95	0.004	−0.009 to 0.017	0.55	1.96	1.96	0.000	−0.014 to 0.014	0.99	271	270	0.719	−1.239 to 2.677	0.47	279	278	0.994	−0.995 to 2.983	0.33

Per protocol: days with heater use ≥ 30%^c^	1.91	1.88	0.030	0.003 to 0.057	0.03	1.91	1.91	0.008	−0.021 to 0.037	0.59	269	268	0.832	−3.505 to 5.168	0.71	277	272	5.185	1.032 to 9.338	0.01

Asthma subgroup, ITT analysis^d^	1.84	1.84	0.001	−0.038 to 0.041	0.95	1.84	1.85	−0.017	−0.061 to 0.027	0.45	253	250	2.285	−4.954 to 9.524	0.54	260	253	7.130	0.578 to 13.682	0.03

Asthma subgroup, per-protocol analysis^e^	1.84	1.79	0.056	−0.027 to 0.139	0.19	1.74	1.80	−0.059	−0.174 to 0.056	0.31	255	255	0.021	−11.637 to 11.679	1.00	260	251	8.910	−7.543 to 25.362	0.29

Diff, difference.

a The means and differences and the 95% CIs for the differences are derived from mixed model regression. Fixed effects were the heater type allocation, study period (first, second, or third pair of weeks), and day of the week. Schools and subjects within schools were both treated as random intercepts, and a variance components correlation structure was assumed.

b All observations were analyzed according to the heater type exposure to which they were assigned. There were 6,776 observations for FEV_1_ morning and PEF morning and 6,504 observations for FEV_1_ evening and PEF evening

c The analysis was limited to schools and days for which heater use was estimated as ≥ 30%. There were 1,249 observations for FEV_1_ morning and PEF morning and 1,441 observations for FEV_1_ evening and PEF evening.

d All observations in subjects with current asthma at baseline were analyzed according to the heater type exposure to which they were assigned. There were 982 observations for FEV_1_ morning and PEF morning and 987 observations for FEV_1_ evening and PEF evening.

e The analysis was limited to schools and days for which heater use was estimated as ≥ 30% in subjects with asthma. There were 169 observations for FEV_1_ morning and PEF morning and 213 observations for FEV_1_ evening and PEF evening.

**Table 5:** Prevalence of wheeze and cough by gas heater (flued or unflued) type and population subgroup.

	Morning cough		Evening cough		Morning wheeze		Evening wheeze	
Population	Flued	Unflued	Flued	Unflued	Flued	Unflued	Flued	Unflued
ITT analysis^a^	14.2	14.2	20.6	21.6	4.4	4.9	5.2	5.7
Per-protocol analysis^b^	16.8	15.9	23.2	23.2	5.4	4.4	5.3	5.6
Atopic subgroup, ITT sample^a^	16.3	14.6	21.0	21.9	4.9	6.5	6.9	8.1
Atopic subgroup, per-protocol sample^b^	21.7	17.7	24.9	24.0	5.3	6.8	6.6	9.4

a All observations were analyzed according to the heater type exposure to which they were assigned.

b The analysis was limited to schools and days for which heater use was estimated as ≥ 30%.

**Table 6:** Effect of heater type (unflued gas heater/flued gas heater) on symptoms.

	Morning symptoms^a^		Evening symptoms^b^		Morning cough		Evening cough		Morning wheeze		Evening wheeze		Sore teeth		Stomachache		Use of bronchodilators for relief of symptoms	
Population	OR^c^ (95% CI)	*p-*Value	OR^c^ (95% CI)	*p-*Value	OR^c^ (95% CI)	*p-*Value	OR^c^ (95% CI)	*p-*Value	OR (95% CI)	*p-*Value	OR (95% CI)	*p-*Value	OR (95% CI)	*p-*Value	OR (95% CI)	*p-*Value	OR (95% CI)	*p-*Value
ITT analysis^d^	0.931 (0.810–1.071)	0.32	0.962 (0.859–1.076)	0.49	0.994 (0.850–1.162)	0.94	1.161 (1.008–1.336)	0.04	1.381 (1.041–1.832)	0.03	1.116 (0.856–1.454)	0.42	0.861 (0.630–1.179)	0.35	1.156 (0.894– 1.496)	0.27	0.870 (0.590–1.282)	0.48

Per-protocol analysis^e^	0.737 (0.512–1.060)	0.10	0.888 (0.671–1.176)	0.41	0.872 (0.574–1.325)	0.52	1.192 (0.842 –1.686)	0.32	1.577 (0.640–3.888)	0.32	1.582 (0.714–3.507)	0.26	1.357 (0.584–3.149)	0.48	0.993 (0.513–1.923)	0.98	0.843 (0.273– 2.602)	0.77

ITT analysis, adjusted for home use of gas, open fire for heating, and exposure to ETS^f^	0.938 (0.801–1.098)	0.43	0.963 (0.852–1.089)	0.55	1.006 (0.844–1.200)	0.94	1.103 (0.949–1.283)	0.20	1.603 (1.171–2.194)	0.003	1.123 (0.856–1.473)	0.40	0.897 (0.644–1.249)	0.52	1.309 (0.997–1.718)	0.05	0.890 (0.596–1.329)	0.57

Atopic subgroup, ITT sample^g^	0.805 (0.650–0.997)	0.05	0.920 (0.764–1.107)	0.38	0.769 (0.604–0.979)	0.03	1.156 (0.923–1.448)	0.21	1.849 (1.258–2.718)	0.002	1.144 (0.814–1.607)	0.44	0.846 (0.524–1.365)	0.49	1.627 (1.064–2.488)	0.02	1.868 (1.075–3.247)	0.03

Atopic subgroup, per-protocol sample^h^	0.843 (0.498–1.427)	0.52	0.958 (0.594–1.544)	0.86	0.722 (0.399–1.306)	0.28	1.256 (0.718–2.197)	0.42	7.721 (1.551– 38.434)	0.01	3.968 (1.369–11.502)	0.01	1.427 (0.436–4.674)	0.56	1.311 (0.444– 3.867)	0.62	7.040 (0.945–52.442)	0.06

a Reported either cough or wheeze in the morning.

b Reported cough, wheeze or chest tightness, fever, sore throat, runny or blocked nose, eye irritation, sore teeth or gums, or stomachache in the evening.

c ORs > 1 indicate that exposure to the unflued gas heater, compared with the flued gas heater, is associated with an increased risk of symptoms. The model used to estimate these ORs is described in “Methods.”

d All observations were analyzed according to the heater type exposure to which they were assigned. There were 9,079 observations for morning wheeze and 8,858 observations for evening wheeze.

e The analysis was limited to schools and days for which heater use was estimated as ≥ 30%. There were 1,668 observations for morning wheeze and 1,986 observations for evening wheeze.

f ITT analysis adjusted for home use of gas heating, open fire, and exposure to ETS; all observations were analyzed according to the heater type exposure to which they were assigned. The additional time-variable covariates were included in the model. There were 7,179 observations for morning wheeze and 7,604 observations for evening wheeze.

g ITT analysis limited to subjects with atopy. There were 3,418 observations for morning wheeze and 3,320 observations for evening wheeze.

h Per-protocol analysis limited to subjects with atopy. There were 596 observations for morning wheeze and 706 observations for evening wheeze.
